# Growth traits, hematological, and ruminal fluid profile of sheep offered ensiled coffee skin replacing dried water spinach

**DOI:** 10.14202/vetworld.2023.1238-1245

**Published:** 2023-06-08

**Authors:** Amam Amam, Mochammad Wildan Jadmiko, Pradiptya Ayu Harsitax, Osfar Sjofjan, Danung Nur Adli

**Affiliations:** 1 Department of Animal Science, Faculty of Agriculture, Universitas Jember, East Java, Indonesia; 2 Department of Feed and Animal Nutrition, Faculty of Animal Science, Universitas Brawijaya, Malang, East Java, Indonesia

**Keywords:** biochemical blood, coffee skin, crossbreed sheep, ensiling, final body weight

## Abstract

**Background and Aim:**

Developing simple, cost-efficient sheep feed will improve farmers’ incomes. Including coffee skin in feed offers the most technical method of increasing sheep weight gain. This study aimed to evaluate varying proportions of ensiled coffee skin replacing dried water spinach and determine the optimal combination for the growth performance, physiological and hematological profiles, and rumen fluid of sheep.

**Materials and Methods:**

Eighty-four animals were randomly allocated to the treatments, arranged in a randomized block design using the initial weight as a block. Seven treatment diets were adjusted and a 12-animal replication was used for each treatment. The treatments were as follows: T0: 30% maize stover, 30% dried water spinach, 5% pollard, 20% coffee skin; T1: 30% maize stover, 25% dried water spinach, 5% pollard, 5% ensiled coffee skin; T2: 30% maize stover, 20% dried water spinach, 5% pollard, 10% ensiled coffee skin; T3: 30% maize stover, 15% dried water spinach, 5% pollard, 15% ensiled coffee skin; T4: 30% maize stover, 10% dried water spinach, 5% pollard, 20% ensiled coffee skin; T5: 30% maize stover, 5% dried water spinach, 5% pollard, 25% ensiled coffee skin; T6: 30% maize stover, 5% pollard, and 30% ensiled coffee skin. The sheep were reared for 70 days.The parameters observed during the early stage included growth performance (initial body weight, LW gain, final body weight, and feed intake). At the end of periods, a representative sample of ruminal fluid (approximately 150 mL) was collected from slaughtered sheep, duplicated, and then incubated for 18 h and blood samples were collected from the sheep (jugular vein) in ethylenediaminetetraacetic acid tubes. Then, used to analyze various blood biochemical parameters.

**Results:**

The final body weights showed a linear curve increasing as the treatment increased (p < 0.05). The ensiled coffee skin tended to increase at 6 h incubation time, producing reduced methane gas (p < 0.05). However, in general, the use of ensiled coffee skin did not significantly alter the blood biochemistry of crossbreed sheep (p > 0.05). There was no significant effect on the protozoal population (p > 0.05).

**Conclusion:**

Increasing the level of ensiled coffee skin up to 30% replacing dried water spinach increased the final body weight of crossbreed sheep with no adverse effect.

## Introduction

Sheep are a type of livestock that can help meet national protein consumption. Up to the third quarter of 2022, Indonesia’s national population was 17,902,911 heads of sheep [1]. Surprisingly, Indonesia has started exporting up to 60,000 heads of lamb every year. The increasing need for national meat consumption is dependent on the following three factors: (1) Breeding; (2) feeding; and (3) management and health. To support this condition, the government and industry sectors have sought to defend the national sheep stock by effectively utilizing feeds. Improved feeding regimes are essential to meet the nutrient requirements of ruminants in the tropics. Indonesian farmers largely continue to employ cut-and-carry systems. Developing simple, cost-efficient sheep feed will improve farmers’ incomes. Including coffee skin in feed offers the most technical method of increasing sheep weight gain. Indonesia’s national coffee production stood at 774,60 thousand tons at the end of 2022, up from 762,20 thousand tons at the end of 2021 (an increase of 1.62%). The production in East Java Province reached 48,675 tons [1]. It was reported by Oliveira and Franca [1] that although coffee is one of the top agricultural products, the total coffee produced generates waste of approximately 30–50%. At the same time, the national production of water spinach was only 38.480 tons at the end of 2022. The use of water spinach offered to sheep was favorable among the local farmer in Indonesia. Furthermore, the nutrient content between water spinach and coffee skin was quite similar in crude fiber (CF), crude protein (CP), neutral detergent fiber (NDF), and acid detergent fiber (ADF) [2].

Coffee skin without ensiling in sheep has been found to make an insignificant difference to sheep’s growth performance or blood profile [2]. This may be attributable to the anti-nutritional factors in coffee skin, such as caffeine and tannin [2]. Another study by San Martin *et al* [3]. reported that using coffee agricultural by-products did not affect performance. Indonesia comprises tropical areas with high-temperature fluctuations and high humidity, resulting in a high-stress level and discomfort. In such conditions, there are limitations in terms of the water that can be provided for grassed areas. In addition, the production of water spinach was unstable due to tropical conditions and limitations of the water. The ensiling technique is both favorable and technically possible due to its low cost and appropriateness for smallholder farmers in Indonesia [4, 5]. The hematological and physiological profiles of sheep are influenced by various factors, including breed, age, environmental conditions, related heat stress, and transportation. Furthermore, tannins have been described to treat hematological profiles. However, study on the inhibitory effects of tannin related to hematological profile and gene expression are not yet understood [5].

In the context of a lack of information on the use of ensiled coffee skin, this study aimed to evaluate the use of varying proportions of ensiled coffee skin replacing dried water spinach and to determine the optimal combinations for growth performance, physiological and hematological profiles, and the rumen fluid of sheep to improve fattening systems.

## Materials and Methods

### Ethical approval

This study was approved by the Animal Ethics Committee of Universitas Brawijaya KEP. 12/09/2022.

### Study period and location

The study was conducted from September to December 2022 at PT. Gumuk Mas Farm, Jember in East Java Province, Indonesia (Latitude −8° 19’ 4.7568”, Longitude 113° 24’ 0.7776”).

### Animals

Eighty-four crossbreed fat-tailed sheep × indigenous local sheep were used (from 3 to 4 months of age) with a mean live weight (LW) of 18.2 kg. The animals were purchased from the local animal market and individuals were identified using ear tags. The feed was adapted over 14-day adaptation periods during the pre-experiment adaptation period. The sheep were reared for 70 days. Eighty-four male sheep were randomly allocated to the treatments and arranged in a randomized block design using their initial weight as a block. Seven treatment diets were compiled using a 12-animal replication for each treatment. The treatments used were as follows: T0: 30% maize stover, 30% dried water spinach, 5% pollard, 20% coffee skin; T1: 30% maize stover, 25% dried water spinach, 5% pollard, 5% ensiled coffee skin; T2: 30% maize stover, 20% dried water spinach, 5% pollard, 10% ensiled coffee skin; T3: 30% maize stover, 15% dried water spinach, 5% pollard, 15% ensiled coffee skin; T4: 30% maize stover, 10% dried water spinach, 5% pollard, 20% ensiled coffee skin; T5: 30% maize stover, 5% dried water spinach, 5% pollard, 25% ensiled coffee skin; T6: 30% maize stover, 5% pollard, and 30% ensiled coffee skin (Table-1). The parameters observed during the early stage included growth performance (initial body weight, LW gain, final body weight, and feed intake). A representative sample of ensiled coffee skin was taken and analyzed for its dry matter (DM), organic matter (OM), CP, total digestible nutrient (TDN), ether extract (EE), CF, NDF, ADF, ash, and tannin following the method described by AOAC [4] (Tables-1 and 2) while the formulated feed was following the method described by BSN [6].

**Table-1:** Ingredients proportion and chemical compositions in the experimental diets (% on DM basis) of crossbreed sheep.

Ingredient	Treatments (%)						

	T0	T1	T2	T3	T4	T5	T6
Maize stover	30	30	30	30	30	30	30
Dried water spinach	30	25	20	15	10	5	-
Copra meal	5	5	5	5	5	5	5
Maize gluten feed	5	5	5	5	5	5	5
Dried distillers grain soluble	3	3	3	3	3	3	3
Pollard	5	5	5	5	5	5	5
Molasses	2	2	2	2	2	2	2
Coffee skin	20	-	-	-	-	-	-
Ensiling coffee skin	-	5	10	15	20	25	30
DM, (%) DM	34.35	43.23	34.56	33.32	34.21	35.22	34.57
OM, (%) DM	84.32	85.42	86.21	87.21	85.65	88.67	87.22
CP, (%) DM	11.01	11.45	11.53	11.56	12.31	12.01	12.11
TDN, (%) DM	72.31	76.22	75.23	74.25	75.23	76.22	76.55
EE, (%) DM	3.34	2.67	2.84	2.89	3.21	3.33	3.22
CF, (%) DM	19.26	18.91	17.65	18.05	19.05	18.05	18.66
NDF, (%) DM	40.87	41.25	42.33	41.25	40.11	41.23	41.44
ADF, (%) DM	30.82	31.88	30.82	31.35	32.44	30.22	31.22
Total Tannin	8.28	7.39	7.31	7.33	7.25	7.22	7.21

DM=Dry matter, ADF=Acid detergent fiber, CP=Crude protein, CF=Crude fiber, DM=Dry matter, EE=Ether extract, NDF=Neutral detergent fiber, TDN=Total digestible nutrient

**Table-2:** Nutrient content in the experimental diets (% on DM basis) of crossbreed sheep.

Composition	Ingredients								

	Maize Stover	Dried water spinach	Pollard	Copra meal	Molasses	DDGS	Maize gluten feed	Coffee skin	Ensiling coffee skin
DM (%)	28.3	82.33	88.4	87.1	74.0	89	90	89.12	86.00
CP (%)	9.2	6.23	12.45	6.5	2.3	10.25	7.85	6.78	8.02
TDN (%)	76.54	71.23	76.34	73.2	79.23	78.25	74.25	72.35	75.35
EE (%)	1.3	1.43	5.1	12.1	7.4	5	3	2.45	3.00
CF (%)	23.2	25.44	8.23	18.7	0.2	7	1.5	27.00	28.70
Ash (%)	6.5	5.67	6.4	5.6	0.2	5.66	5.55	7.93	6.87
NDF (%)	68.20	42.33	35.65	56.2	57.89	25.62	23.44	39.03	41.22
ADF (%)	39.20	37.81	9.41	20.3	60.23	8.90	10.24	26.63	25.44
Tannin (%)	-	-	-	-	-	-	-	4.71	3.45

ADF=Acid detergent fiber, CP=Crude protein, CF=Crude fiber, DDGS=Dried distillers grain soluble, DM=Dry matter, EE=Ether extract, NDF=Neutral detergent fiber, TDN=Total digestible nutrient

### Feeding programs and preparation of coffee skin for ensiling

The coffee skin for ensiling was obtained from a local coffee farmer in the Jember area. Briefly, 30 kg (wet basis) was freshly collected, and then immediately processed for ensiling. *Saccharomyces cerevisiae* was obtained commercially from (Rapid, Universitas Brawijaya). *Saccharomyces cerevisiae* was chosen to increase the nutrient composition of coffee skin by promoting a rapid fermentation-producing protein and breakdown of sugar. The use of *S. cerevisiae* increased aerobic stability. The ensiling procedure was followed [7, 8] procedures for 30 days. First, the fresh coffee skin was weighed; second, it was placed on a tray and combined with the following *S. cerevisiae* levels: 0%; 0.2%; 0.4%; 0.6%; 0.8%; 1.0%; and 1.2%. The *S. cerevisiae* was chosen from 0% to 1.2% since the additive can be given not more than 1.5%. Third, the samples were placed in a silo and transferred to the fermentation station room under anaerobic and room temperature at 25°C–28°C (Table-3). Fourth, the silo was opened after 30 days and the distraction was removed from the surface of the samples. At the same time, the water spinach was dried in the sun drying for 3 days before being given to the sheep. The feeds were offered in a sequence of 3 times/day at 08.00 h, 13.00 h, and 16.00 h. Water was provided *ad libitum* during the experimental period. Feed intake was recorded daily by measuring the amount of feed offered and refused. All of the sheep were weighed individually in the morning before feeding using a weighing scale (True Test, New Zealand). The LW gain was expressed as the difference between the final and initial weights of the sheep divided by the day conducted.

**Table-3:** Temperature and relatively humidity during experimental.

Parameter	September	October	November	December	SEM
Temperature (°C) morning	27.3	27.5	26.5	26.4	0.32
RH (%) morning	80.3	81.0	80.25	79.25	0.56
Temperature (°C) afternoon	29.73	28.35	27.35	28.30	0.45
RH (%) afternoon	74.6	73.45	72.35	71.25	0.34

RH=Relatively humidity, SEM=Standard error mean

### Biochemical blood analyses

Blood samples were collected from the sheep (jugular vein) and in ethylenediaminetetraacetic acid tubes. A total 20 mL plasma was immediately centrifuged using a cryogenic centrifuge (Hettich Universal 320 R, Germany) at 704.34× *g* for 15 min. The samples were kept in tubes at −20°C until chemical analysis and used to analyze various biochemical parameters [9]. These included hemoglobin, packed cell volume (PCV), hematocrit, erythrocytes, leukocytes, mean corpuscular volume, mean corpuscular hemoglobin, mean corpuscular hemoglobin concentration, platelet, heterophils, lymphocytes, monocytes, eosinophils, basophils, glucose, cholesterol, and low-density lipoprotein.

### Ruminal fluid profile and gas production kinetics

A total of 250 mg ground test treatment (Wiley Mill, 2 mm) into 5–50 mL glass vials. A buffer solution (synthetic saliva) was prepared. A representative sample of ruminal fluid (approximately 150 mL) was collected from slaughtered sheep, duplicated, and then incubated for 18 h. Next, a formaldehyde liquid solution and formal saline (0.8% NaCl) were added to obtain a total protozoan count. This was percolated through a four-layer cloth, warmed at 38°C and centrifuged at 1.126× *g* for 10 min. The strained ruminal inoculate was mixed thoroughly at 39°C with synthetic saliva (1:2 v/v) to obtain a homogenous digestion medium. A homogenous digestion medium (25 mL) was added into glass vials for each treatment. The vials were placed in a shaker and the gas production test involved 2, 4, 6, 8, 12, 24, 48, 72, and 96 h of incubation. The total volatile fatty acid (VFA) was incubated for 12 h and approximately 100 mL solution was distilled and titrated using phenolphthalein liquid with 0.05 NaOH solution.

### Statistical analysis

Before the statistical analysis, procedure analysis of variance (ANOVA) using a one-way ANOVA nested design was carried out using SAS OnDemand for Academics (ODA, Cary, NC, USA). The results are presented as the standard error of the mean. Moreover, the differences between means were calculated using the least significant difference testing. The following model was used [10, 11]:


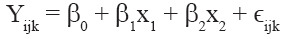


Where Y_ij_ is the parameters observed, β_0_ is the overall mean, β_1_x_1_ the effect level coffee skin, β_2_x_2_ the ensiled coffee skin and ϵ_ijk_ the error number. The difference was significant (p < 0.05) or not significant (p > 0.05). Moreover, probability values were calculated using Duncan testing, to determine any significant difference (p < 0.05). The production of gas was calculated using the following model [12]:


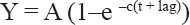


Where Y is the total gas production at time t, A is the gas produced between soluble and insoluble fractions, c is the constant rate (%/h) of gas production from the total insoluble fraction, t is the incubation time (h), and lag is the lag phase time (h).

## Results

The temperature in the morning was higher in October at 27.5°C while, afternoon was higher in September at 29.73° (Table-3). In addition, the relative humidity was high in October at 81% (Table-3). The final body weight and DM digestibility were depicted by a linear curve that increased as the level of treatment increased (p < 0.05) (Table-4). It is, therefore, not surprising that the level of ensiled coffee skin did not produce a significant difference in the remainder of the growth traits such as LW gain, DM intake, and OM digestibility (p > 0.05) (Table-4). The ensiled coffee skin produced a significant difference in PCV (p < 0.05) but did not present a significant difference across whole hematological of crossbreed sheep (Table-5). However, in general, the use of ensiled coffee skin did not produce a significant difference in the blood biochemistry of the crossbreed sheep (p > 0.05) (Table-6). The level of ensiled coffee skin tended to increase at 6 h of incubation time. It produced a reducing amount of methane gas (p < 0.05) while, at the rest of the time did not present a significant difference (p > 0.05) (Table-7). At the same time, increased ensiled coffee skin produced a significant difference in asymptotic gas production (p < 0.05) but did not present significant differences in fractional rate of fermentation and lag phase of fermentation (p > 0.05). There was no significant effect on the rumen fluid pH, pH of feces, ammonia, and protozoal population (p > 0.05) (Table-8). The level of ensiled coffee skin produced a significant difference in the percentage of CH_4_ (p < 0.05) (Table-8).

**Table-4:** Effects of the ensiling coffee skin on growth traits and nutrient digestibility of crossbreed sheep.

Parameter	Treatments (%)							

	T0	T1	T2	T3	T4	T5	T6	SEM
IW (kg)	18.30	19.00	19.12	19.13	19.25	19.20	18.75	0.12
FBW (kg)	30.24^a^	30.7^ab^	32.59^b^	33.43^b^	34.94^b^	36.00^bc^	36.30^bc^	3.12
LWG (g)	324.24	365.24	421.64	440.24	324.25	323.21	436.25	0.09
DMI (g/day)	870.23	882.12	842.11	810.22	832.22	894.22	833.22	0.07
FCR	8.01	8.02	8.21	7.54	9.85	9.03	8.00	0.25
DMD	59.22^a^	59.34^a^	60.51^ab^	62.03^b^	63.21^b^	62.31^b^	61.11^ab^	1.58
OMD	64.35	63.42	62.33	64.51	63.22	64.55	62.31	1.22

DMD=Dry matter digestibility, DMI=Dry matter intake, FBW=Final body weight, FCR=Feed conversion ratio, g=Gram, Kg=Kilogram, IW=Initial weight, LWG=Live weight gain, OMD=Organic matter digestibility, SEM=Standard error mean. ^a, b, c, d^ Means with different superscripts in the row differ significant (p<0.05)

**Table-5:** Effects of the ensiling coffee skin on hematological of crossbreed sheep.

Parameter	T0	T1	T2	T3	T4	T5	T6	SEM
Hemoglobin (g/dL)	12.51	11.21	11.03	13.21	10.21	12.12	11.22	0.32
PCV (%)	31.98^b^	28.87^ab^	30.12^b^	28.64^a^	27.66^a^	27.56^a^	27.77^a^	0.21
Hematocrit (%)	32.15	31.40	34.22	33.12	35.21	32.01	33.15	0.21
Erythrocytes (×10^6^/mm^3^)	2.23	2.95	2.51	2.13	2.62	2.21	2.33	0.48
Leukocytes (×10^6^/mm^3^)	2.23	2.32	2.41	2.54	2.56	2.15	2.17	0.23
MCV (fL)	105.25	131.0	134.65	126.81	126.15	124.12	125.22	21.20
MCH (pg)	33.2	34.1	34.6	35.2	33.6	37.2	32.0	0.76
MCHC (%)	30.2	33.2	31.3	32.1	37.6	35.4	33.2	0.23
PLT (×10^6^/mm^3^)	15.2	14.3	16.2	15.2	12.3	11.3	12.1	1.78
Heterophils (×10^9^/dm^3^)	3.2	4.3	2.3	2.5	2.1	3.1	2.3	0.13
Lymphocytes (×10^9^/dm^3^)	2.34	2.33	2.32	1.87	1.83	1.82	4.2	1.18
Monocytes (×10^9^/dm^3^)	0.03	0.03	0.04	0.07	0.07	0.06	0.08	0.004
Eosinophils (×10^9^/dm^3^)	1.00	1.02	1.03	0.87	0.88	0.78	0.67	0.02
Basophils (×10^9^/dm^3^)	1.01	0.76	0.83	0.84	0.76	0.78	0.67	0.35
Heterophils (%)	32	43	23	25	21	31	23	1.16
Lymphocytes (%)	53.2	55.25	56.75	54.25	55.1	54.34	56.23	2.23
Monocytes (%)	2.5	2.3	2.6	2.5	2.3	2.5	2.4	0.12
Eosinophils (%)	2.34	2.54	2.33	2.33	2.22	1.87	1.98	0.47
Basophils (%)	0.6	0.7	0.7	0.5	0.6	0.32	0.6	0.27

fL=Femtoliters, dL=Deciliters, g=Gram, MCH=Mean corpuscular hemoglobin, MCHC=Mean corpuscular hemoglobin concentration, MCV=Mean corpuscular volume, PCV=Packed cell volume, PLT=Platelet, SEM=Standard error mean, ^ab^Mean within a row with different superscripts present significantly different p*<*0.05

**Table-6:** Effects of the ensiling coffee skin on blood biochemistry of crossbreed sheep.

Parameter	T0	T1	T2	T3	T4	T5	T6	SEM
Glucose (mg/dL)	44.23	45.21	44.35	46.25	44.51	43.25	45.25	1.33
Total cholesterol (mg/dL)	43.29	44.31	45.32	45.21	43.21	45.12	43.87	2.35
LDL (mg/dL)	34.22	35.23	37.23	34.13	34.22	35.65	32.32	3.55

LDL=Low-density lipoprotein, dL=Deciliters, mg=Milligram, SEM=Standard error mean

**Table-7:** Effects of the ensiling coffee skin on gas production of crossbreed sheep.

Incubation hour	T0	T1	T2	T3	T4	T5	T6	SEM
2	26.50	26.39	26.76	26.54	26.53	26.43	25.60	0.76
4	56.32	54.22	57.23	55.23	54.24	55.23	56.25	2.34
6	75.65^a^	87.22^b^	89.25^bc^	90.22^c^	88.23^b^	89.25^bc^	91.22^c^	0.67
8	92.33^a^	101.25^b^	110.25^bc^	115.25^c^	120.33^c^	122.35^c^	123.56^c^	1.37
12	131.23	135.25	136.25	134.24	135.22	136.21	134.22	1.22
24	150.22^a^	176.23^bc^	178.23^bc^	181.24^c^	185.25^c^	191.75^d^	195.25^d^	2.08
48	210.33^a^	225.65^b^	235.65^bc^	240.22^bc^	256.25^c^	265.24^c^	280.22^cd^	2.16
72	285.23	286.22	287.33	285.22	283.24	283.25	281.25	2.18
96	285.34	286.54	287.22	288.22	286.52	283.32	281.22	3.11
A, mL/g DM	230.11^a^	235.22^b^	241.67^bc^	245.35^c^	246.87^c^	247.85^c^	248.34^c^	1.67
C, h	0.103	0.087	0.056	0.023	0.211	0.268	0.256	0.03
Lag, h	0.78	0.67	0.54	0.66	0.67	0.78	0.72	0.34

DM=Dry matter, A=Asymptotic gas production, C=Fractional rate of fermentation, Lag=Lag phase of fermentation, SEM=Standard error mean. ^abc^Mean within a row with different superscripts present significantly different p*<*0.05

**Table-8:** Effects of the ensiling coffee skin on ruminal fluid profile and methane gas of crossbreed sheep.

Parameter	T0	T1	T2	T3	T4	T5	T6	SEM
Rumen fluid pH	6.8	6.7	6.7	6.7	6.7	6.6	6.6	0.07
pH faeces	6.8	6.7	6.6	6.5	6.4	6.4	6.7	0.04
Ammonia (mg N-NH_3_/dL)	8.2	9.0	9.1	8.4	8.3	8.2	8.1	0.18
Protozoa (×10^4^) mL	0.7	0.6	0.5	0.4	0.4	0.4	0.5	0.16
VFA (Mm)								
Acetate (C_2_)	50.5	54.3	53.3	52.5	55.2	56.25	54.32	0.14
Propionate (C_3_)	48.2	41.55	42.50	42.15	43.11	42.11	45.15	0.13
Butyrate (C_4_)	20.3	21.3	20.4	21.4	20.5	20.5	20.1	0.33
C_2_/C_3_ ratio	1.22	1.14	1.45	1.33	1.18	1.12	1.32	0.34
CH_4_ (%)	2.34^c^	2.02^bc^	1.76^b^	1.54^ab^	1.43^a^	1.52^ab^	1.44^a^	0.22

mg=Milligram, SEM=Standard error mean, VFA=Volatile fatty acid. ^abc^Mean within a row with different superscripts present significantly different p*<*0.05

## Discussion

### Effects of ensiled coffee skin on the growth traits of crossbred sheep

The presented result was compared with that reported for the use of ensiled *Alternanthera brasiliana*, which was voluntarily increased in sheep. At the same time, DM intake, final body weight and average daily gain increased linearly after offering ensiled *A. brasiliana*. The result of the study may be associated with high levels of CP and TDN and sits linearly with [13]’s feeding of less protein required for microbial rumen, which caused a reduction in LW gain and FBW. The linear increase in microbial activity was correlated with the total population in the ruminal fluid. The present study reveals that the increase in LW gain and FBW of sheep after being offered ensiled coffee skin may be explained by its high protein content and TDN. In contrast, the relatively low CP concentration in the coffee skin may be attributable to the harvesting time and fluctuating weather conditions. At the same time, the decline of CP depends on the increasing maturity. In addition, Marley *et al*. [14] revealed that a combination of ensiled red clover and Lucerne resulted in a significant difference compared with the use of ensiled kale. Several factors affected the results of the ensiling process of the materials.

The higher pH indicated a lower concentration of lactic acid bacteria in the forage and more restricted fermentation during the ensiling process [14]. During the ensiling process, several factors indicate the presence of an enzyme and oxidative processes, which inhibit proteolysis in either the silo or the rumen. Interestingly, the final body weight of the sheep fed on ensiled coffee skin was higher than those not fed with ensiled coffee skin, despite the sheep fed on coffee skin silage having a similar weight to the control treatment during the experimental period. Overall, the sheep were adapted for LW prior, this finding was dependent on the sheep fed ensiled coffee skin being heavier than those fed un-ensiled coffee skin at the end of the adaptation period. This indicates that the sheep offered ensiled coffee skin adapted more readily to the change in coffee skin silage. In another result, Niayale *et al*. [15] reported that using cassava from agro-industrial by-products offered to sheep under tropical conditions presented a significant difference in both the final weight and LW gain compared with fresh and dried. During harvesting time, tropical agro-industrial by-products mostly have a lower water-soluble carbohydrate level and high cell walls. As a result, *Bacillus* microorganisms are much more undesirable than lactic acid bacteria, which are efficient during the conversion of water-soluble carbohydrates. In addition, besides the ensiling process, physical appearance is influenced by the acceptability preferences of sheep when offered ensiled forages [16]. Contrary to the finding from Jamal and Abdallah [17], feeding sheep with agro-industrial by-products produced an insignificant difference in either the final body weight gain or LW gain. These results may be due to the prevalence of anaerobic conditions suitable for developing lactic acid bacteria that are late to form [17].

### Effects of ensiled coffee skin on the hematological profile of crossbred sheep

In line with Mako *et al*. [18], the use of ensiled *A. brasiliana* was revealed to be significant in terms of hemoglobin and PCV. Packed cell volume is representative of measuring toxicity in the blood, whereby a low value indicates anemia. Lower oxygen capacity in the blood causes animal anemia [19]. Ibhaze *et al*. [20] reported that feeding various foliage and maize stover presented a significant difference. This may be due to the small number of anti-nutritional factors in the blood. The remainder of the hematological profiles showed no significant difference from this result after offering ensiled coffee skin. However, Amuda and Okunlola presented the contrary findings [21]; here, while an insignificant difference was reported in PCV, there was also a tendency for cholesterol and glucose to decrease after offering ensiled maize stover. Amuda and Okunlola [21] reported that the lymphocytes in small ruminants such as sheep should be in the range of 34.00%–51.00%, while PCV should be 22.50%–30.00% for normal small ruminants. Furthermore, cholesterol and glucose levels are normally within the ranges of 50.00–140.00 mg/dL and 55.0–131.00 mg/dL, respectively [21]. A higher cholesterol mean value indicates a low-fat content in the feed [21].

### Effects of ensiled coffee skin on gas production, ruminal fluid, and methane gas in crossbred sheep

The amount of gas produced in the rumen is representative and indicates the amount of feed that sheep can digest. A study by Sadarman *et al*. [5] reported that ensiled agro-industrial by-products with no additives showed no significant difference. Contrary findings showed that adding up to 2% additive produced a significant difference. Carbohydrates and glucose are the major contributors to gas production. Gas production would appear to be associated with the substrate [8]. Numerous VFAs are found in the rumen: acetic acid, butyrate acid, and propionic acid. The reduction of methane gas in Sadarman *et al*. [8] showed no correlation with the microorganism used but was correlated with the amount of tannin added to the total mixed ratio. Adding tannin to the agro-industrial by-product ensilage led to the microorganism producing proteolytic enzymes to increase the protein content. The amount of gas produced depends on the maturity of the forages [22]. While the presence of anti-nutritional factors such as tannin and saponin reduces the level of gas production as they act as inhibitory agents on ruminal microorganisms [23, 24]. In line with this, components such as starch and non-structural carbohydrates, CP, neutral detergent fiber, and acid detergent fiber led to differences in gas production [23]. The presence of tannins in sheep was mainly impacted by the animal age, amount of tannin in the diet, and covariates groups [25].

In addition, Pagés-Díaz and Huiliñir [26] mentioned that pH and VFAs depended on various factors, including moisture and particle length. The proportion of VFAs reflected the high carbohydrate content of feed material, which provides propionate; however, this was also dependent on the carbohydrate level [27]. In addition, the amount of gas produced depends on several carbohydrate substances such as sugars, pectin, and starch [8]. These three carbohydrate fractions lead to rapid gas production during the early hours of incubation. In contrast, during ensiling, stimulants such as molasses, enzymes, and the lactic acid bacteria group help to increase the fermentation process [23, 28]. Lactic acid bacteria such as *Lactobacillus plantarum*, *Lactobacillus* *acidophilus*, *Pediococcus acidilactici*, *Pediococcus* *pentacaceus*, and *Enterococcus faecium* create a lower pH environment and have been found to reduce pH below 4, thus preventing further degradation of the sugar or protein in the silage [23, 29, 30].

In light of the previous findings, gas production also correlates with DM and OM digestibility [8]. Furthermore, ammonia-N is a by-product of the proteolytic degradation of protein, which is undesirable in the ensiling process [15]. Rumen microorganisms have been shown to begin hydrolyzing and deaminating CP from feed into ammonia-N and peptides [31]. It has also been shown that the concentration of ammonia-N in the rumen represents the balance between hydrolysis and protein utilization in the rumen [24]. The number of VFAs also depends on the DM and CP content [22]. For example, Ribeiro *et al*. [22] demonstrated that when ensiling, immature swards can reduce the production of methane gas as the lower fiber content shifts from fermentation to propionate production [33, 34]. The ability of tannins influenced to reduce rumen protozoa population affected rumen defaunation [25]. Rumen defaunation has been demonstrated to reduce the nutrient digestibility of OM [32].

## Conclusion

Crossbreed sheep offered alternative agro- industrial by-products, notably ensiled coffee skin, which had a higher final body weight than those offered un-ensiled products. Therefore, in addition to limiting the greenhouse effect, local farmers can apply the technique accordingly. These findings demonstrate the potential for using agro-industrial by-products as alternative feed sources, with ensiled coffee skin has shown to improve the nutrient efficiency and small-scale sustainability of sheep fattening systems. Further work is currently being undertaken to determine the economic and environmental impact of the nutrient budget plan for small-scale sheep holdings in Jember.

## Authors’ Contributions

AA: Sample collection, *in vivo* test, writing-review and editing, and investigation. MWJ: Sample collection and *in vitro* test. PAH: Sample collection and *in vivo* test, writing-review and editing, and investigation. OS: Conceptualizations, methodology, sample collection and *in vitro* test, validation, and writing the original draft. DNA: Conceptualizations, formal analysis, software, and writing-original draft. All authors have read, reviewed, and approved the final manuscript.

## References

[1] Oliveira L.S, Franca A.S (2015). An overview of the potential uses for coffee husks. Coffee in Health and Disease Prevention.

[2] Hernández-Bautista J, Rodríguez-Magadán H.M, Villegas-Sánchez J.A, Salinas-Rios T, Ortiz-Muñoz I.Y, Aquino-Cleto M, Lozano-Trejo S (2018). Health status and productivity of sheep fed coffee pulp during fattening. Austral J. Vet. Sci.

[3] San Martin D, Orive M, Iñarra B, García A, Goiri I, Atxaerandio R, Urkisa J, Zufia J (2021). Spent coffee ground as second-generation feedstuff for dairy cattle. Biomass Conv. Bioref.

[4] AOAC (2000). Association of Official Analytical Chemists.

[5] Sadarman I.A, Ridla M, Jayanegara A, Nahrowi Ridwan R, Sofyan A, Herdian H, Darma I.N.G, Wahyono T, Febrina D, Harahap R.P, Nurfitriani R.A, Adli D.N (2022). Influence of ensiling and tannins addition on rumen degradation kinetics of soy sauce residues. Adv. Anim. Vet. Sci.

[6] BSN (2019). Standard National of Indonesia (SNI) 8819, Feed for Sheep.

[7] Sjofjan O, Adli D.N, Natsir M.H, Nuningtyas Y.F, Bastomi I, Amalia F.R (2021). The effect of increasing levels of palm kernel meal containing a-b-mannanase replacing maize to growing-finishing hybrid duck on growth performance, nutrient digestibility, carcass trait, and VFA. J. Indones. Trop. Anim. Agric.

[8] Sadarman S, Ridla M, Nahrowi N, Ridwan R, Jayanegara A (2020). Evaluation of ensiled soy sauce by-product combined with several additives as an animal feed. Vet. World.

[9] Rathwa S.D, Vasava A.A, Pathan M.M, Madhira S.P, Patel Y.G, Pande A.M (2017). Effect of season on physiological, biochemical, hormonal, and oxidative stress parameters of indigenous sheep. Vet. World.

[10] Adli D.N, Sjofjan O, Irawan A, Utama D.T, Sholikin M.M, Nurdianti R.R, Nurfitriani R.A, Hidayat C, Jayanegara A, Sadarman S (2022). Effects of fiber-rich ingredient levels on goose growth performance, blood profile, foie gras quality and its fatty acid profile:A meta-analysis. J. Anim. Feed Sci.

[11] Ardiansyah W, Sjofjan O, Widodo E, Suyadi S, Adli D.N (2022). Effects of combinations of a-*Lactobacillus* spp. and *Curcuma longa* flour on production, egg quality, and intestinal profile of Mojosari ducks. Adv. Anim. Vet. Sci.

[12] Amani-Yengejeh M.M, Taghizadeh A, Mohammadzadeh H, Hosseinkhani A, Shirmohammadi S, Abachi S, Giannenas I (2022). Utilization of slow-release non-protein nitrogen produced from agro-industrial by-products:Feed digestibility and ruminal parameters. J. Anim. Feed Sci.

[13] Rajabi R, Tahmasbi R, Dayani O, Khezri A (2017). Chemical composition of alfalfa silage with waste date and its feeding effect on ruminal fermentation characteristics and microbial protein synthesis in sheep. J. Anim. Physiol. Anim. Nutr. (Berl).

[14] Marley C.L, Fychan A.R, Fraser M.D, Sanderson R, Jones R (2007). Effects of feeding different ensiled forages on the productivity and nutrient-use efficiency of finishing lambs. Grass Forage Sci.

[15] Niayale R, Addah W, Ayantunde A.A (2020). Effects of ensiling cassava peels on some fermentation characteristics and growth performance of sheep on-farm. Ghana J. Agric. Sci.

[16] Ososanya T.O, Olorunnisomo O.A (2015). Silage characteristics and preference of sheep for wet brewer's grain ensiled with maize cob. Livest. Res. Rural Dev.

[17] Jamal A.B.O, Abdallah J (2019). Performance of Assaf lambs fed two upgraded agricultural wastes. Walailak J. Sci. Technol.

[18] Mako A.A, Ikusika O.O, Akinmoladun O.F (2021). Physiological response of WAD sheep fed different combinations of Guinea grass and ensiled *Alternanthera brasiliana* (L.) O. Kuntze based diets:Intake, haematology and serum biochemical indices. Vet. Anim. Sci.

[19] Jiwuba P.C, Ikwunze K, Ume S.I, Nsidinanya N.O (2016). Performance, apparent nutrient digestibility and cost benefit of West African dwarf goats fed dietary levels of *Moringa oleifera* leaf meal. J. Adv. Biol. Biotechnol.

[20] Ibhaze G.A, Ogunjemite G.E, Fajemisin A.N (2021). Blood chemistry of West African dwarf goats fed treated maize cob-and maize husk-based diets with mixture of microorganisms. Bull. Natl. Res. Cent.

[21] Amuda A.J, Okunlola D.O (2018). Hematological parameters and serum biochemistry of West African dwarf sheep fed ensiled maize Stover and concentrate supplements. J. Agric. Vet. Sci.

[22] Ribeiro G.O, Teixeira A.M, Velasco F.O, Faria W.G, Jayme D.G, Maurício R.M, McAllister T.A (2015). Methane production and energy partitioning in sheep fed *Andropogon gayanus* grass ensiled at three regrowth stages. Can. J. Anim. Sci.

[23] Chaji M, Direkvandi E, Salem A.Z (2020). Ensiling of *Conocarpus erectus* tree leaves with molasses, exogenous enzyme and *Lactobacillus plantarum* impacts on ruminal sheep biogases production and fermentation. Agrofor. Syst.

[24] Alipour D, Rouzbehan Y (2007). Effects of ensiling grape pomace and addition of polyethylene glycol on *in vitro* gas production and microbial biomass yield. Anim. Feed Sci. Technol.

[25] Torres R.N.S, Ghedini C.P, Paschoaloto J.R, da Silva D.A.V, Coelho L.M, Almeida G.A, Almeida M.T.C (2022). Effects of tannins supplementation to sheep diets on their performance, carcass parameters and meat fatty acid profile:A meta-analysis study. Small Rumin. Res.

[26] Pagés-Díaz J, Huiliñir C (2020). Valorization of the liquid fraction of co-hydrothermal carbonization of mixed biomass by anaerobic digestion:Effect of the substrate to inoculum ratio and hydrochar addition. Bioresour. Technol.

[27] Yanegh A, Ghanbari F, BayatKouhsar J, Moslemipur xF (2019). Chemical composition and gas production parameters of tomato pomace ensiled with sugar beet pulp and bacterial inoculation and its effect on the performance of Moghani fattening lambs. Anim. Sci. J.

[28] Kholif A.E, Gouda G.A, Morsy T.A, Matloup O.H, Fahmy M, Gomaa A.S, Patra A.K (2022). Dietary date palm leaves ensiled with fibrolytic enzymes decreased methane production, and improved feed degradability and fermentation kinetics in a ruminal *in vitro* system. Waste Biomass Valor.

[29] Hillion M.L, Moscoviz R, Trably E, Leblanc Y, Bernet N, Torrijos M, Escudié R (2018). Co-ensiling as a new technique for long-term storage of agro-industrial waste with low sugar content prior to anaerobic digestion. Waste Manag.

[30] Özelçam H, İpçak H.H, Özüretmen S, Canbolat Ö (2021). Feed value of dried and ensiled *Paulownia* (*Paulownia* spp.) leaves and their relationship to rumen fermentation, *in vitro* digestibility, and gas production characteristics. Rev. Bras. Zootec.

[31] Symeou S, Tsiafoulis C.G, Gerothanassis I.P, Miltiadou D, Tzamaloukas O (2019). Nuclear magnetic resonance screening of changes in fatty acid and cholesterol content of ovine milk induced by ensiled olive cake inclusion in Chios sheep diets. Small Rumin. Res.

[32] Newbold C.J, De la Fuente G, Belanche A, Ramos-Morales E, McEwan N.R (2015). The role of ciliate protozoa in the rumen. Front. Microbiol.

[33] Fajemisin A.M, Fadiyimu A.A, Mokan J.A (2010). Performance and nitrogen retention in West African Dwarf goats fed sun dried *Musa sapiertum* peel and *Gliricidia sepium*. J. Trop. Agric.

[34] Taha V.J, Huntington J, Wilkinson R, Davies D (2022). The effect of supplemented chestnut tannin to grass silage either at ensiling or at feeding on lamb performance, carcass characteristics and meat quality. Agric. Food Sci.

